# Fabrication and Characterization of an Innovative Silver- and Gadolinium-Doped Bioglass for Bone Regeneration

**DOI:** 10.7759/cureus.51086

**Published:** 2023-12-25

**Authors:** Hari Vamsh Korukonda, Nidhita Suresh, Saranya K

**Affiliations:** 1 Dentistry, Saveetha Dental College and Hospitals, Saveetha Institute of Medical and Technical Sciences, Saveetha University, Chennai, IND; 2 Periodontology, Saveetha Dental College and Hospitals, Saveetha Institute of Medical and Technical Sciences, Saveetha University, Chennai, IND

**Keywords:** silver, gadolinium, bone regeneration, quality of life, bioglass

## Abstract

Background

Periodontal regeneration aims for the three-dimensional reconstruction of bone defects, and over the years, bone grafts with or without barrier membranes have provided us with promising results. Particulate bone grafts can be classified according to the source of procurement as autografts, allografts, xenografts, and alloplasts. Bioglass, an innovative alloplast that uses silica particles as a matrix incorporated with calcium and phosphorus, has been extensively used as a propitious material for bone regeneration owing to its inherent osteogenic ability and biocompatibility but presents with various disadvantages such as slow degradation rate, low mechanical strength, and fracture resistance. A novel silver (Ag)-gadolinium (Gd)-doped bioglass was fabricated to improve the mechanical properties, biocompatibility, and osteogenic ability when compared with bioglass (control).

Materials and methods

The Ag- and Gd-doped bioglass network was prepared and assessed for the morphological and structural properties by scanning electron microscopy (SEM) analysis, X-ray diffraction (XRD), and attenuated total reflectance (ATR)-infrared (IR). The cytotoxicity of Gd and Ag-doped bioglass was assessed using the MG63 cell line through the MTT (3-(4,5-dimethylthiazol-2-yl)-2,5 diphenyl tetrazolium bromide) assay at various concentrations and the absorbance of the solution was measured at 570 nm using a microplate reader. The osteogenic ability of the material was assessed by alkaline phosphatase activity and collagen estimation.

Results

ATR-IR spectroscopy, SEM, and XRD were used to examine the bioglass network doped with Gd and Ag. ATR-IR exhibited classic silicate bands, whereas SEM indicated particles bigger than 5 μm. XRD analysis revealed the production of Na2Ca2Si3O9, Na2Ca4(PO4)2SiO4, and wollastonite. The excellent crystallinity of Na2Ca2Si3O9 provided the bioglass network with good mechanical characteristics.

The Gd-Ag-bioglass did not exhibit any toxicity towards the living cells at increasing concentrations from 12.5 µg to 100 µg. The alkaline phosphatase activity was increased by 10% and the collagen estimation remained consistent with bioglass (control).

Conclusion

In conclusion, the fabrication of the novel Gd-Ag-doped bioglass shows good cytocompatibility and osteogenic ability and shows great potential to enhance bone regeneration.

## Introduction

Larry Hench invented bioactive glass in 1969. This glass has been used as the earliest bioactive material and is considered an innovative material that stimulates a beneficial response from the body and bone to the host tissue, which is usually bone [1]. Bioglass is hypothesized as osteoinductive because it bonds strongly with the host bone by dissolving to form an apatite layer on the bone surface. The dissolution products of the bioglass, including silica and calcium ions, stimulate the cells to create a bone matrix [2]. Despite its uniqueness and applications in bone tissue engineering over the years, bioglass has short fracture toughness and mechanical properties [3], limiting its application in load-bearing areas. Its structure is more porous than the cancellous structure of human bone [4,5]. To counteract these disadvantages and improve its mechanical properties, we have fabricated an innovative gadolinium (Gd)-silver (Ag)-doped bioglass.

Incorporating silver into this bioglass network is advantageous for silver’s antibacterial and potential osteoinductive properties. It achieves osteoinductivity by increasing the proliferation and differentiation of mesenchymal stem cells (MSCs). The differentiation and proliferation of MSCs were induced by the silver nanoparticles’ activation of the TGF- β/BMP signaling pathways. Ag also enhances bone regeneration by acting as a chemoattractant for MSCs and fibroblasts [6].

In recent years, additions of rare earth (RE) elements such as Gd have direct or indirect effects on bone cells and bone minerals, altering the homeostasis of bone circulation to treat bone mineral density disorders, supervising the continuous bone remodeling cycle, and influencing bone formation and resorption [7]. The distinctive antimicrobial, antibacterial, anti-inflammatory, and biocompatibility properties of these emerging rare earth elements have captivated the attention of researchers, who are now exploring their use in tissue engineering. Researchers have developed a variety of RE smart nanobiomaterials for bone tissue engineering. Gd also has been added to magnesium alloys to increase their mechanical properties [8]. Bioglass has been modified with various other materials such as graphene oxide and Ag to alter the osteogenic, angiogenic, and antibacterial efficacy of the bioglass [9,10]. This is the first study to combine Ag and Gd. In this study, we fabricated an innovative composite to counteract the pitfalls of bioglass and assessed for its biocompatibility and osteogenic efficacy.

## Materials and methods

Ag and Gd were doped onto the bioglass network using the sol-gel method and the structure and morphology of the composite were assessed using scanning electron microscopy (SEM) and Fourier transform infrared spectroscopy (FTIR). The biocompatibility was assessed using the MTT (3-(4,5-dimethylthiazol-2-yl)-2,5 diphenyl tetrazolium bromide) assay and the osteogenic ability of the material was determined by alkaline phosphatase activity and collagen estimation.

Synthesis of Ag- and Gd-doped bioglass network

The bioglass network doped with Ag and Gd was prepared by adding 0.45 molarity of tetraethyl ortho silicate, 10 ml of ethanol, 10 ml of double-distilled water, and 2 ml of nitric acid. The mixture was stirred for 24 hours until it formed a gel-like structure. Once the resultant mixture formed into a gel-like consistency, 0.245 moles of calcium nitrate, 0.6 moles of orthophosphoric acid, 0.235 moles of sodium hydroxide, 0.05 moles of Gd nitrate, and 0.05 moles of Ag nitrate were added. The mixture was then stirred for three hours and placed in a hot air oven for 100⁰C and then shifted to a muffle furnace at 70⁰C and the final mixture was ground.

Characterization of the Gd-Ag bioglass composite 

The bioglass network, incorporating Ag and Gd, was assessed for its morphology through SEM (field emission SEM (FESEM), JEOL JSM IT800, JEOL Ltd., Tokyo, Japan). Additionally, an energy-dispersive X-ray (EDX) analysis was employed to detect various biomaterials present in the Gd-Ag-bioglass composite. To investigate the chemical composition of the bioglass network, FTIR spectra were recorded using a Bruker Alpha II instrument with attenuated total reflectance (ATR) sampling (Bruker, Billerica, Massachusetts, United States). Furthermore, X-ray diffraction (XRD) analysis was carried out on the sintered specimen, both before and after immersion in simulated body fluid (SBF). A CuK radiation source operating at 40 kV and 40 mA was used in the analysis, and the resulting diffraction patterns were recorded in the 2θ range spanning from 10 to 75 degrees [11].

Biocompatibility of the Gd-Ag-bioglass composite

In the Gd-Ag-bioglass cytotoxicity assessment, the MG63 cell line was utilized in the MTT assay. Cells were seeded into 96-well plates containing varying concentrations of bioglass (12.5, 25, 50, 75, and 100 g/mL) and then incubated for 24 hours. Following the established experimental protocol found in the literature, the solution’s absorbance was measured at 570 nm using a microplate reader to assess cellular metabolic activity and potential toxicity.

Alkaline phosphatase activity

Upon reaching cell confluence, samples were introduced and left to incubate for three days. Following this, cells were solubilized using Triton X-100 (Dow Chemical Company, Midland, Michigan, United States) and incubated for one minute. To assess alkaline phosphatase (AP) activity and measure protein production, an AP buffer was introduced. After 5-bromo-4-chloro-3-indolyl phosphate (BCIP)/nitro blue tetrazolium (NBT) was added and the samples were incubated for 30 minutes, cell density was measured at 405 nm using an enzyme-linked immunosorbent assay (ELISA) plate reader, and fluorescence microscope images were captured. Protein content was determined using the Bradford assay, which was optimized for accuracy. The samples were transferred to a new 96-well plate post-BCIP and NBT treatment, and both AP buffer and Bradford reagent were added. Optical density was then measured at 595 nm, and normalization was achieved by dividing the optical density by the cell count at each culture time.

Collagen estimation

Cells were first placed in a 96-well plate, permitting them to adhere and attain confluence over a 24-hour incubation. Following this, the culture medium was substituted with a medium containing 2% fetal calf serum, along with the introduction of the test compound. Subsequently, cells were subjected to an additional 48-hour incubation at 37°C with both control and treated cells receiving medium replacements every 24 hours.

Subsequent to the incubation phase, the cells underwent a series of steps. They were first rinsed with saline then harvested and immersed in 4% formalin for 20 minutes. Following fixation, the cells experienced three cycles of washing with phosphate-buffered saline (PBS) before being subjected to a 20-minute Sirius red staining process at 37°C with 20 mL of staining solution. Subsequently, three additional PBS washes were carried out, followed by treatment with 10% acetic acid. Afterward, the cells underwent another three PBS washes and were subsequently stained with picrosirius red for a duration of one hour. The final stages included washing the samples with acidified water, dehydrating them with ethanol, and visualizing them under a fluorescent microscope.

## Results

**Figure 1:**
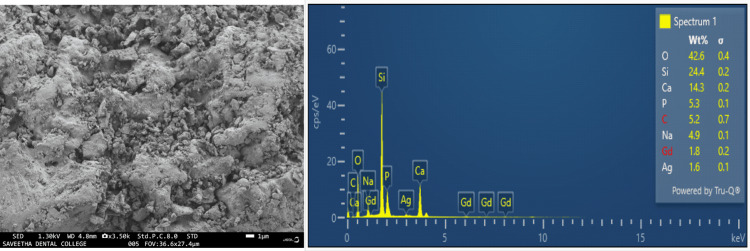
SEM analysis and EDX spectra of the Gd-Ag-bioglass SEM: scanning electron microscopy; EDX: energy-dispersive X-ray; Gd: gadolinium; Ag: silver; O: oxygen; Si: silicon; Ca: calcium; P: phosphorus; C: carbon; Na: sodium

The SEM examination revealed a predominantly coarse texture in the bioglass network, with particles generally exceeding 5 µm in size. However, it is worth noting the existence of a small fraction of finer powders, as illustrated in Figure 1. The EDX spectra for the innovative Gd-Ag-bioglass demonstrated the surface composition of the sample, featuring 1.8% Gd, 1.6% Ag, 24.4% silicon (Si), 14.3% calcium (Ca), and 5.3% phosphorus (P), all presented in weight percentage, as depicted in Figure 1.

**Figure 2:**
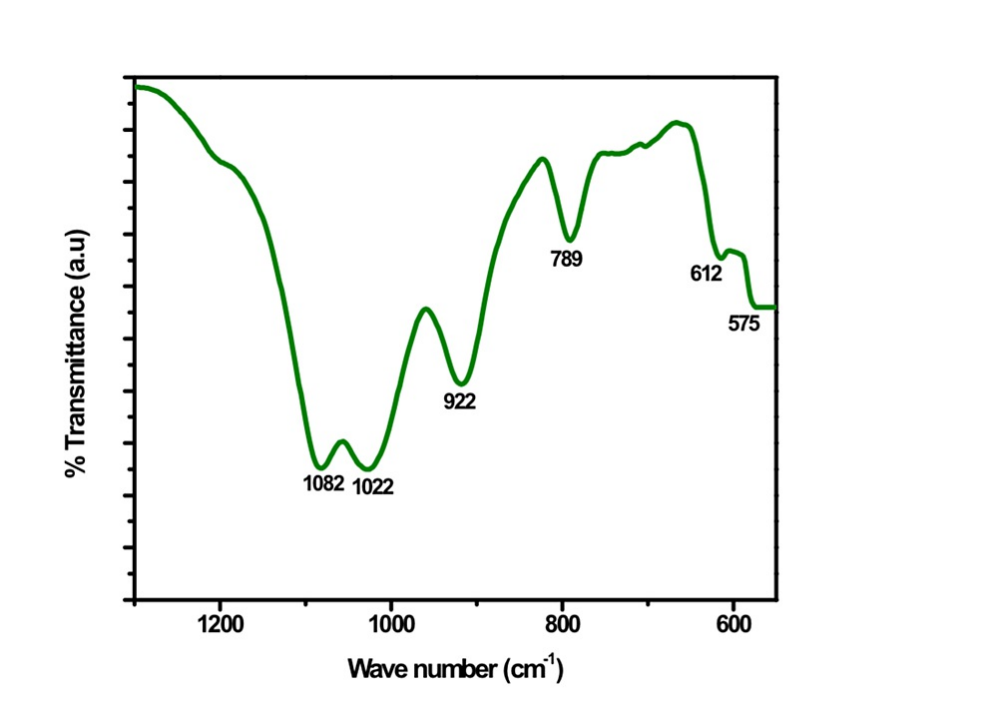
ATR-IR spectrum of Gd-Ag-bioglass ATR-IR: attenuated total reflectance-infrared; Gd: gadolinium; Ag: silver

The attenuated total reflectance (ATR)-infrared (IR) spectroscopy assessment of the Gd-Ag-bioglass network, as depicted in Figure 2, demonstrated the presence of characteristic silicate bands consistent across all spectra. These bands were associated with vibration peaks at 1085 and 789 cm-1, denoting the asymmetric stretching mode and symmetric stretching vibration of Si-O-Si, respectively [11,12]. Moreover, the presence of specific peaks in the spectra was observed. The peak at 922 cm^-1^ was indicative of the P-O-P stretching occurring within the prepared bioglass network. Additionally, the peak at 1022 cm-1 corresponds to the antisymmetric stretching modes of Si-O-Si tetrahedra, which involve the bridging oxygen. A smaller peak observed at 575 cm^-1^ is attributed to P-O bonds, suggesting a crystalline apatite-like calcium phosphate P-O bending mode. Last, a peak at 612 cm^-1^ is associated with the symmetric stretching of P-O vibrations in the crystalline silicate, as outlined in Table 1 [13].

**Table 1:** Wave numbers observed from the ATR-IR spectra ATR-IR: attenuated total reflectance-infrared; Si: silicon; O: oxygen; P: phosphorous

Wave number (cm^-1^)	
1120	Asymmetric stretching of Si-O-Si
1042	Anti-symmetric stretching modes of Si-O-Si tetrahedra within the bridging oxygen
922	P-O-P stretching symmetric. Si-O-Si stretching vibration
612	P-O vibrations
575	P-O bending mode

**Figure 3:**
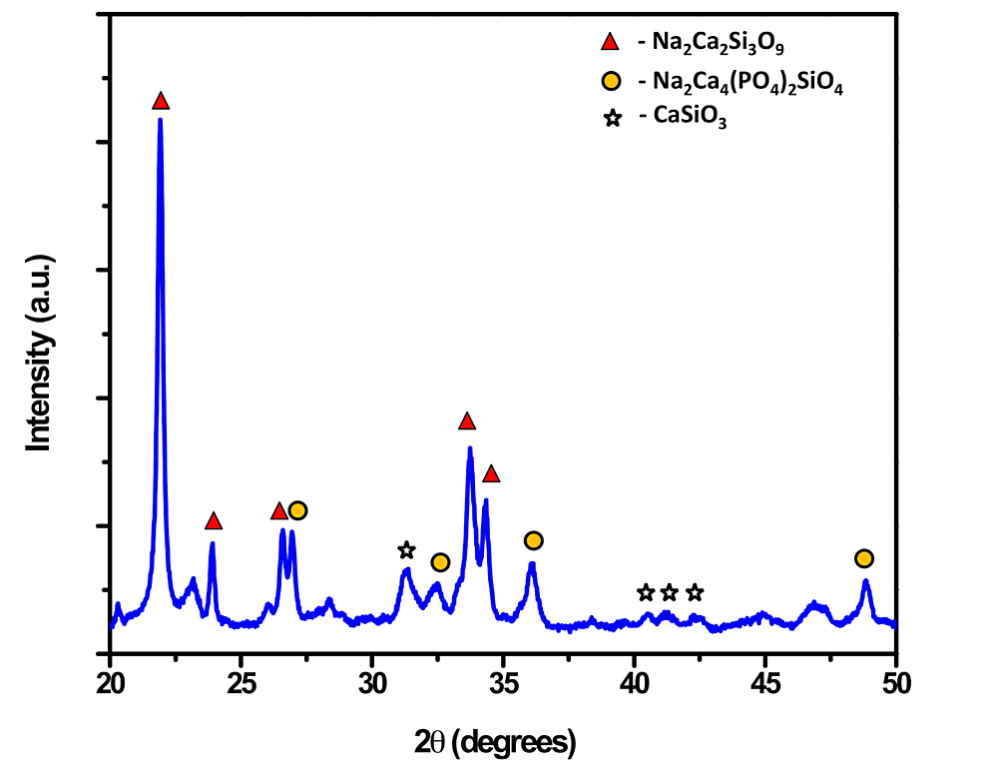
XRD analysis of the Gd-Ag-bioglass XRD: X-ray diffraction; Gd: gadolinium; Ag: silver

The XRD analysis (Figure 3) revealed a strong correspondence with the standard Powder Diffraction File (PDF) #22.1455, confirming the formation of crystalline phases including Na2Ca2Si3O9 (PDF #075-1687), Na2Ca4(PO4)2SiO4 (PDF #032-1053), and Wollastonite (CaSiO3; PDF #84-0655). Notably, the bioglass network exhibited excellent mechanical properties attributable to the extensive densification and finely crystalline nature of Na2Ca2Si3O9.

**Figure 4:**
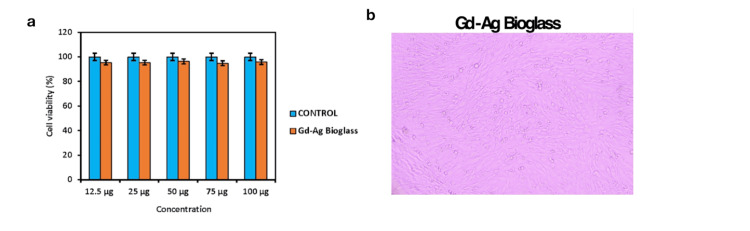
a-Quantification of the cell viability % between the control and Gd-Ag-bioglass, b- Microscopic image of the cell viability in Gd-Ag-bioglass Gd: gandolinium; Ag: silver

The experimental results indicated that the Gd-Ag-bioglass exhibited nearly identical cell viability when compared with the normal bioglass. The cellular viability percentages were consistent between the two bioglass types at different concentrations ranging from 12.5 µg to 100 µg as shown in Figures 4a, 4b. This study revealed that the Gd-Ag-bioglass displayed no toxicity toward living cells. These findings strongly suggest that the Gd-Ag-bioglass holds promise for biomedical applications given its non-toxic and biocompatible characteristics.

**Figure 5:**
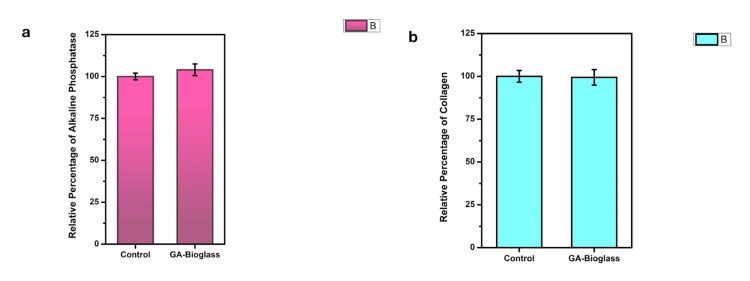
a-Quantification of alkaline phosphatase stained with (BCIP/NBT) for Gd-Ag-bioglass and control (bioglass), b-Collagen stained with alizarin red for Gd-Ag-bioglass and control (Bioglass) BCIP/NBT: 5-bromo-4-chloro-3-indolyl phosphate/nitro blue tetrazolium; Gd: gandolinium; Ag: silver

The relative percentage of alkaline phosphatase activity was measured in cells treated with two types of bioglass: the control (normal bioglass) and the Gd-Ag-bioglass. The results show that the Gd-Ag-bioglass exhibited higher alkaline phosphatase activity than the control bioglass at all tested concentrations (50%, 75%, 100%, and 125%), as shown in Figure 5a. The collagen estimation results indicated that both the Gd-Ag-bioglass and the control (normal bioglass) had nearly identical collagen content. Across various concentrations (25%, 50%, 75%, 100%, and 125%), the relative collagen percentages were consistent between the two bioglass types. This implies that the inclusion of Ag and Gd had minimal influence on collagen content, suggesting similar potential for bone regeneration in both bioglass variations, as shown in Figure 5b.

## Discussion

Periodontal therapy’s foremost goals are to halt the disease’s progression, prevent recurrence, and restore destroyed periodontal structures. There is a constant search for an ideal material to regenerate the periodontal tissues lost due to disease [14]. Bioglass has been proven promising with its osteoconductive and osteostimulation behavior in periodontal regeneration alone or in combination with other biomaterials [15-19]. Abushahba et al., in their systematic review, have concluded that bioglass is effective in treating periodontal bone defects [20]. However, the material has many disadvantages, such as delayed degradation rate, low mechanical strength, and fracture resistance. We have crafted an innovative composite of bioglass consisting of Gd and Ag. This composite serves a dual purpose: it can be employed as a particulate bone graft or seamlessly integrated into scaffolds to augment the outcomes of periodontal tissue engineering. The comprehensive understanding of the interaction of bioactive glass within a physiological context and its impact on bone formation has emerged from extensive research spanning decades in which this material has been utilized as a bone graft. The glass’s ability to dissolve gradually upon exposure to water stands as a fundamental characteristic underpinning these effects. Following implantation, bioactive glass disperses and releases soluble ions originating from its composition. These ions, in turn, trigger a bioactive response, leading to the formation of a hydroxycarbonate apatite (HCA) layer on the glass’s surface [21].

The bioactive glass has been shown to boost both osteoconduction and osteostimulation properties, facilitating bone formation. Its angiogenic effects have even been noted in some research, including increased vascular endothelial growth factor (VEGF) release, increased VEGF gene expression in fibroblasts, increased endothelial cell proliferation, and the development of endothelial tubules in vitro. Additionally, it has demonstrated the capacity to enhance vascularization in vivo [22]. The purpose behind creating this bioglass network was to harness the combined benefits of Ag and Gd to enhance the graft’s structural and morphological characteristics. Examination through SEM images revealed that the particles within the fabricated bioglass network exceeded 5 µm in size. This size is of significance as it promotes rapid protein and cell adhesion, facilitates cell migration, and supports osseointegration [23]. The ATR-IR spectra patterns closely resemble those of hydroxyapatite, suggesting a transformation of the initially amorphous structure of the bioglass powder into crystalline hydroxyapatite [24].

The integration of Ag into the bioglass network effectively tackles potential infections that may occur during guided tissue regeneration (GTR). GTR entails the use of barrier membranes that could potentially become surfaces for biofilm formation. This becomes particularly concerning when non-resorbable membranes are employed, as they might be susceptible to colonization by microbial pathogens if prematurely exposed during surgical placement [25]. However, to combat such issues, the use of graft materials and membranes coated with antibiotics have been used widely along with systemic antibiotics. In the present research, we have fabricated the bioglass network doped with silver to provide for non-cytotoxic antibacterial activity, thereby enhancing osteoblast cell lineage cell growth and bone regeneration [26]. Silver nanoparticles have also undergone evaluation for their toxicity and cytocompatibility, revealing their reduced toxicity to the embryonic development of zebrafish [27]. Silver nanoparticles enhance osteogenic potential through various pathways, including the differentiation and proliferation of MSCs [6], upregulation of osteogenic protein expression, and promotion of mineralization in human MSCs via autophagy regulation [28]. Balamurugan et al. confirmed the antibacterial activity and its effectiveness in periodontal pockets under anaerobic conditions of the bioglass doped with bioglass due to the leaching of silver ions from the bioglass [10].

The inclusion of Gd in the bioglass network contributes positively to the regulation of cell behavior and bone regeneration. Gd is typically found accumulated in human bones [29]. Gd3+ ions within the bioglass network stimulate the increased expression of p-Smad1/5/8, thereby expediting the osteogenic differentiation of rat bone marrow MSCS (rBMSCs) through the Smad/Runx2 signaling pathway [30]. Gd along the cerium has the ability to scavenge reactive oxygen species (ROS) at the cellular and histological levels [31]. Elevated production of ROS occurs as a defensive response in cases of periodontal disease. This increased ROS production can result in the degradation of periodontal structures [32]. ROSs have the potential to significantly expedite the formation of new bone and the deposition of collagen fibers. The current study has its constraints such as that the degradation profile, mechanical property, and osteogenic ability of the complex have not been evaluated, and hence, future research should be done on in vitro and in vivo models with animal and human participants to analyze the efficiency of this Ag-Gd-doped bioglass in bone regeneration.

## Conclusions

In conclusion, research into the production and characterization of Ag- and Gd-doped bioglass for bone regeneration is a promising topic that may result in the creation of new biomaterials with enhanced capabilities for bone healing. The development of this novel Ag-Gd-doped bioglass complex can be used as an effective biomaterial in periodontal regeneration. Further in-vitro and in-vivo studies would be necessary to incorporate this complex into clinical situations.
